# Supplementary material for: Antioxidants Supplementation Reduces Ceramide Synthesis Improving the Cardiac Insulin Transduction Pathway in a Rodent Model of Obesity
Source: Nutrients. 2021 Sep 28;13(10):3413. doi: 10.3390/nu13103413 (PMC8541644; doi:10.3390/nu13103413)
Supplement: Supplementary file 1 [file nutrients-13-03413-s001.zip › nutrients-1376681-supplementary.pdf]

Table S1. Final body weight of experimental rodents as well as plasma glucose and insulin levels and homeostatic model assessment for insulin resistance (HOMA-IR) after n-acetylcysteine (NAC) administration in rats fed a standard diet (Control) or high-fat diet (HFD). The data are expressed as mean values ± SD and are based on ten independent determinations (n = 10). * $p < 0.05$ significant difference vs. corresponding Control group; # $p < 0.05$ significant difference vs. corresponding High-Fat Diet group.

**Parameters**	**Control**	**HFD**	**NAC**	**HFD + NAC**
Body weight (g)	282.8 ± 15.0	350.3 ± 14.7 *	270.3 ± 14.6 #	320.5 ± 9.0 *,#
Glucose (mg/dl)	115.5 ± 0.7	127.0 ± 4.2 *	107.5 ± 9.9 #	109.0 ± 8.9 #
Insulin (μIU/ml)	14.7 ± 1.8	29.2 ± 4.2 *	20.0 ± 4.7 *,#	25.5 ± 2.5 *,#
HOMA-IR	4.4 ± 0.6	7.9 ± 0.4 *	4.5 ± 2.0 #	7.1 ± 0.5 *,#

Table S2. Final body weight of experimental rodents as well as plasma glucose and insulin levels and homeostatic model assessment for insulin resistance (HOMA-IR) after α-lipoic acid (ALA) administration in rats fed a standard diet (Control) or high-fat diet (HFD). The data are expressed as mean values ± SD and are based on ten independent determinations (n = 10). * $p < 0.05$ significant difference vs. corresponding Control group; # $p < 0.05$ significant difference vs. corresponding High-Fat Diet group.

**Parameters**	**Control**	**HFD**	**ALA**	**HFD + ALA**
Body weight (g)	313.0 ± 18.5	419.3 ± 24.2 *	307.6 ± 11.9 #	371.5 ± 22.5 *,#
Glucose (mg/dl)	120.7 ± 5.0	131.7 ± 8.3 *	114.3 ± 12.4 #	118.5 ± 7.2 #
Insulin (μIU/ml)	16.6 ± 3.4	30.8 ± 2.3 *	16.2 ± 2.5 #	25.3 ± 1.0 *,#
HOMA-IR	5.3 ± 1.0	9.9 ± 0.5 *	5.2 ± 0.7 #	8.4 ± 0.8 *,#
